# Co-occurrence of resistance to different antibiotics among aquatic bacteria

**DOI:** 10.1186/1471-2180-12-225

**Published:** 2012-10-02

**Authors:** Veiko Voolaid, Arvi Jõers, Veljo Kisand, Tanel Tenson

**Affiliations:** 1 University of Tartu Institute of Technology, Nooruse St 1, Tartu, 50411, Estonia

**Keywords:** Multiresistance, Natural environment, Fresh water

## Abstract

**Background:**

Antibiotic resistance is not confined to pathogens, but is also widespread in various natural environments. In nature the microbes producing antibiotic compounds have been around for millions of years. Heavy use of antibiotics in medicine and veterinary practice may lead to the accumulation of resistance genes in microbial populations, followed by a rise in multiresistant bacteria.

**Results:**

To test the extent of resistance among aquatic bacteria, we have collected 760 isolates resistant to at least one antibiotic. The phylogeny of the isolates covers a wide range of Proteobacteria, Actinobacteria and Bacteroidetes. In order to determine the extent of multiresistance, the isolates were tested on six antibiotics. As the growth rate of the different bacteria was highly variable, the classical medical resistance tests could not be used, and an alternative method considering the full growth curve was developed. In general, the overall resistances to different antibiotics could be explained by random, independent distribution. An exception to this was the resistances against tetracycline and chloramphenicol, which tended to occur in pairs.

**Conclusions:**

We conclude that there is no massive spread of multiresistance determinants in the studied environment, although some specific cases can be found, awaiting for molecular characterization of the resistance mechanisms.

## Background

Antibiotic resistance (AR) among pathogens is an increasing problem for medical and veterinary treatment. During the last decades the number of AR infections has been on the rise, and this trend will certainly continue [1]. The vast majority of antibiotic classes currently used originate from natural compounds, and bacteria have been evolving in the antibiotic-containing natural environment for millions of years [2]. Indeed, AR genes can be detected in sediments that are thousands of years old, millennia before any modern medicine [3]. During the years of medical and veterinary usage of antibiotics, some of the drugs have been constantly escaping into the environment, creating an additional selection pressure for resistance [4]. As expected, AR bacteria can be found in both pristine and anthropogenically influenced environments at relatively high frequencies [5-10]. The common ways of spreading AR include accumulation of mutations in genes already present in the genome, and acquisition of new genes by horizontal gene transfer.

Pathogenic organisms can be multiresistant i.e. they can be insensitive to several antibiotics. This can decrease the chance for successful infection treatment, making it harder and more time consuming. Multiresistance can be facilitated by single proteins like efflux pumps which are able to use several antibiotics as a substrate [11]. Another way of becoming multiresistant is to acquire, by horizontal gene transfer, a plasmid and/or transposon carrying resistance genes for several antibiotics in one cassette [11]. Such plasmids are not uncommon, and over time they can incorporate additional resistance genes [12,13].

Similarly to AR against single antibiotics, multiresistance is not unique to pathogens. Multiresistant organisms have also been found in the natural environment [7,9]. They can be retrieved even from pristine environments that have not been subjected to any direct or obvious pollution by human activity [8,14]. Previous studies looking at antibiotic resistance in the environment have concentrated on specific genera, usually the medically most relevant ones, or on specific resistance determinants [5,7,9,15-17]. Therefore, it is currently not clear how widespread multiresistance is in the environment, or which combinations of resistances tend to occur together.

We chose to analyze AR and multiresistance in a random population of cultivable environmental bacteria from a freshwater river. We did not concentrate on specific genera or other specific groups of bacteria as previous studies have done [5,7,16], but instead used five common antibiotics for the selection of our isolates. All isolates in the collection were tested for resistance against six antibiotics, and the tendencies to multiresistance were estimated.

The difficulties and biases of cultivating environmental bacteria have been well documented [18]. These issues, together with the advances in community DNA-based methods (PCR, sequencing etc.), have directed the field of environmental microbiology away from culture-based approaches [19-21]. On the other hand, it is clear that the current DNA-based methods do not presently allow accurate descriptions to be made of the phenotypes of the bacteria involved, and it is not clear when the new methods will advance to the point of predicting the full array of properties of individual organisms. Therefore, cultivation of antibiotic resistant organisms still provides valuable information. In the current work we have combined cultivation-based methods with molecular approaches to characterize the resistance phenotype and identity of the isolates.

## Methods

### Sampling

Samples from the river Emajõgi in Estonia were taken with a 1.5 liter water sampler. Sampling was carried out at two locations along the river (station 1 – latitude 58°26′4.57"N, longitude 26°39′24.81"E; station 2 – latitude 58°21′30.58"N, longitude 26°53′51.72"E). The sampling was carried out in 4 successive months from July to October 2008. From station 1 the samples were taken on the 21^st^ July, 30^th^ July, 21^st^ August, 11^th^ September and 8^th^ October; the dates were the same for station 2, except in September the sample was taken on the 12^th^. For each sampling, two 0.5 liter replicates were taken from the top of the surface water. The samples were brought to the laboratory within two hours of sampling. Samples were kept at +4°C until further processing.

### Isolation of the study population

Bacteria were isolated by plating 200 μl and 50 μl of samples (in duplicate) on to selective agar plates. Our media contained 80% (v/v) of the collected water sample filtered through GF/F filters (Whatman) and 20% (v/v) distilled water. In addition, 1 g yeast extract, 5 g peptone and 15 g agar (for agar plates) was added per 1 L of medium, after which the medium was autoclaved for 15 min at 121°C. The medium is similar to ZoBell medium [22], but for this study, instead of marine water in ZoBell, fresh water was used. Antibiotics used in the selective media were: ampicillin (100 μg mL^-1^), tetracycline (20 μg mL^-1^), norfloxacin (2 μg mL^-1^), kanamycin (20 μg mL^-1^) and chloramphenicol (30 μg mL^-1^). The plates were incubated at 18°C for up to 72 h. Selection of the study population was based on differences in the morphology of the colonies. From each plate all morphologically different colonies, but not less than 10 per plate, were streaked onto a new plate to be sure to get pure isolates. Pure isolates were grown in liquid media containing the same components as the plates minus the agar. Liquid media contained the antibiotics at the same concentration as used in the agar plates, and the cultures were grown at 18°C for several days, but not longer than 5 days. These cultures were used to extract DNA and make glycerol (15%) stocks which were kept at −86°C.

### DNA extraction

Genomic DNA was extracted from 1.5 mL of liquid culture using the GuSCN-silica method [23]. Briefly, cells from the liquid culture were pelleted by centrifugation at 5,000 rpm for 5 min, the supernatant discarded, and the cell pellet resuspended in 900 μL guanidiniumthiocyanate lysis buffer (5.25 M GuSCN, 100 mM Tris–HCl pH 6.4, 20 mM EDTA, 1.3% Triton X-100) and 20 μl of silica suspension. After resuspending, the mixture was incubated at room temperature for 5 min, then centrifuged at 5,000 rpm for 10 s. The supernatant was discarded and the pellet washed with buffer (5 M GuSCN) and 50% ethanol. The pellet was dried briefly and the nucleic acid was resuspended in 50 μL ultrapure milli-Q water. The sample was stored at −20°C.

### Polymerase chain reaction and sequencing

The complete 16S ribosomal RNA (rRNA) gene was amplified by PCR. Reactions were carried out using the universal primers pcrF (5′-AGAGTTTGATCATGGCTCAG-3′) (positions 6–26 in *E.coli* rDNA), pcrR (5′-TACGGYTACCTTGTTACGACTT-3′) (positions 1513–1492 in *E.coli* rDNA), RupA (5′-CGTATTACCGCGGCTGCT-3′) (positions 536–519 in *E.coli* rDNA) [24]. Each PCR reaction mixture (25 μL) consisted of 12.5 μL PCR Master Mix (2x) (Fermentas Life Sciences), 5 pmol of each primer and approximately 50 ng genomic DNA as the template. The PCR program was the following: initial denaturation for 5 min at 95°C, followed by 30 cycles of denaturation at 94°C for 1 min, annealing at 45°C for 1 min, and extension at 72°C for 2 min, before a final extension at 72°C for 5 min.

The PCR product was purified with MSB® Spin PCRapace (Invitek) and sequenced using an ABI 3130xl Genetic Analyzer. The primers pcrF, pcrR and RupA were used for sequencing the amplified 16S rRNA gene.

### Phylogenetic analysis of the 16S rRNA genes

The 16S rRNA gene sequences were first assembled using Phred and Phrap, followed by editing with Consed [25-27], after which the phylogenetic affiliations were assessed using the Ribosome Database Project [28].

### Determination of antibiotic resistance

To determine the antibiotic resistance, each strain was grown in the liquid medium described above. Six antibiotics at three concentrations were used: ampicillin (10, 25 and 100 μg mL^-1^), meropenem (0.3, 3 and 30 μg mL^-1^), norfloxacin (0.5, 2, 10 μg mL^-1^), chloramphenicol (1, 5, 30 μg mL^-1^), kanamycin (1, 5, 20 μg mL^-1^) and tetracycline (1, 5, 20 μg mL^-1^). The assay was performed in 96-well microtiter plates. The volume of medium in each well was 200 μL. Each well was inoculated with a strain from the 96-well microtiter storage plate using a Liquid Handler: Plate Replicator (V & P Scientific). The plates were incubated at 20°C without shaking. The optical density (OD) was measured at 600 nm after 16, 20, 24, 40 and 64 h. In parallel, the strains were grown on control plates not containing antibiotics.

## Results and discussion

### Isolation of the study population

To study antibiotic resistance in the natural surface water environment we isolated resistant isolates of bacteria from the river Emajõgi. For isolation we used a medium based on the natural water supplemented with peptone and yeast extract. This medium allows a wide phylogenetic and physiological range of water bacteria to be isolated. Previous studies looking at the antibiotic resistant bacteria in freshwater environments have largely used growth media that select for specific phylogenetic or physiological types of bacteria [7,29,30]. The growth medium most similar to the one used by us is Luria-Bertani, which is more nutritious and has been used rarely [31]. Our direct plating approach should allow a wide diversity to be isolated from the community, including rare species. An alternative approach that could be used is prior enrichment of the community members in batch cultures containing only the natural medium i.e. river water, supplemented with antibiotics. However, that method would only enable study of the predominant bacteria, and would miss rare species.

As selective agents five antibiotics were used: ampicillin, chloramphenicol, kanamycin, norfloxacin and tetracycline. These antibiotics were chosen to cover a range of drug targets: DNA replication, protein translation and cell wall synthesis. The antibiotic concentrations were chosen to be greater than or close to the minimum inhibitory concentration (MIC) cutoff values for resistance according to EUCAST [32].

The bacteria were isolated by plating the sampled water directly on to the selective media, followed by incubation at 18°C for several days. The exact incubation period was adjusted according to the growth rate of the colonies. After incubation a set of colonies was selected from each plate and re-streaked several times to obtain pure strains. At least ten colonies were collected from each plate. These colonies were selected to cover the variety of colony morphologies observed. Where there were more than ten morphological types on the plate, the number of collected isolates was increased to include representatives of all the morphotypes.

**Figure 1:**
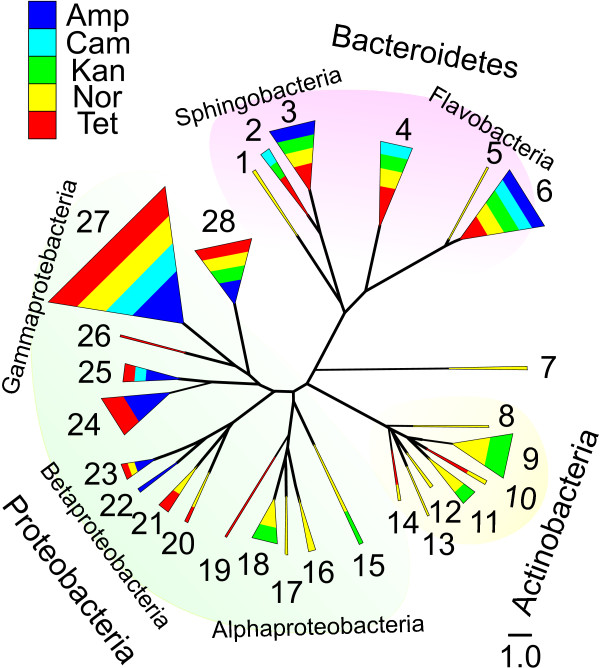
**Unrooted Bayesian phylogenetic tree of the 760 isolates using the 16S rRNA gene sequences.** The scale bar represents 1.0 expected changes per nucleotide position. The nodes are color-coded according to the antibiotics used to isolate the strains, but the area is not proportional to the number of isolates from that antibiotic. The width of the node is in proportion to the number of isolates in each node. The antibiotics are designated as follows: Amp – ampicillin, Cam – chlorapmhenicol, Kan – kanamycin, Nor – norfloxacine, Tet – tetracycline. The numbers indicate genera as follows: 1 – *Flexibacteriaceae*, 2 – *Sphingobacterium*, 3 – *Pedobacter*, 4 – *Flavobacterium*, 5 – *Elizabethkingia*, 6 – *Chryseobacterium*, 7 – *Deinococcus*, 8 – *Brachybacterium*, 9 – *Microbacteriaceae*, 10 – *Cellulomonadaceae*, 11 – *Micrococcaceae*, 12 – *Nocardiaceae*, 13 – *Nocardioidaceae*, 14 – *Sanguibacter*, 15 – *Bacillales*, 16 – *Sphingomonadaceae*, 17 – *Hyphomicrobiaceae*, 18 – *Caulobacteraceae*, 19 – *Ensifer*, 20 – *Alcaligenaceae*, 21 – *Oxalobacteriaceae*, 22 – *Incertia cedis*, 23 – *Comamonadaceae*, 24 – *Aeromonas*, 25 – *Enterobacteriaceae*, 26 – *Acinetobacter*, 27 – *Pseudomonas*, 28 – *Xanthomonadaceae*.

The collection contained 760 isolates. For all of the isolates the 16S rRNA gene was PCR amplified from the genomic DNA and sequenced. The isolates were assembled, using the Ribosome Database Project, according to the 16S rRNA gene sequences, into 9 phylogenetic classes: Actinobacteria, Alphaproteobacteria, Bacilli, Betaproteobacteria, Deinococci, Flavobacteria, Gammaproteobacteria, Sphingobacteria and Thermoprotei (Figure 1). These classes in turn contain representatives of 59 genera. The class containing the largest number of isolates was Gammaproteobacteria, with almost half (49%) of the isolates. More than half (58%) of the Gammaproteobacteria isolates were the 217 strains of *Pseudomonas*. No other genera were represented by more than 100 isolates. The genus *Chryseobacterium* from Flavobacteria and the genus *Stenotrophomonas* from Gammaproteobacteria were the next biggest genera, with 86 and 73 isolates, respectively. Fourteen out of the 59 genera were represented with less than 10 isolates. The phylogenetic composition of the cultivable community isolated in our study in the presence of antibiotics did not differ considerably from the common profile of any aquatic environment [33-35]. The selection towards Gammaproteobacteria is a well known plating bias of aquatic bacterial communities [36]. When the isolates from antibiotic-containing plates were compared with isolates growing on drug free ZoBell medium no striking differences between major genera were observed (Peeter Laas, unpublished data).

We had two sampling stations, one upstream of a town with 100,000 inhabitants (Tartu, Estonia) and the other downstream. No statistically significant differences in the phylogenetic affiliation and AR patterns were observed when bacteria isolated from upstream or downstream were compared (data not shown).

### Characterization of antibiotic resistance

As our isolates showed a wide variety of growth rates and growth curve shapes, the standard MIC test could not be applied. Instead we grew the isolates in 96-well plates in the presence and absence of antibiotic. The cultures were grown at 20°C without shaking and the OD was measured at 16, 20, 24, 40 and 64 h.

All five antibiotics used for the isolation of the strains were used to test the level of resistance of all of the isolates in the collection. As the collection contained a large number of *Pseudomonas* strains, and increased carbapenem resistance is a problem in Estonian medical settings [37], we included a member of this group of antibiotics, meropenem, in the resistance testing.

The growth of an antibiotic-sensitive strain is inhibited by the drug, thus leading to a lower optical density. By dividing the OD value of the test culture containing antibiotic with the control culture without antibiotic we obtained a quantitative value for AR for every timepoint. This value ranged from 0 (no growth in the presence of antibiotic) to 1 (no inhibition by antibiotic), and was used in all subsequent analyses. It is desirable to reduce the AR readings over the time course to a single value characterizing the particular isolate. Therefore, all isolates were characterized by the smallest resistance value over the time course of growth.

We tested all antibiotics at three concentrations, thereby producing three values of AR for each isolate. We presumed that the antibiotic concentration leading to the biggest variability in AR values between the isolates would be the most informative for characterizing the resistance levels in the population. To evaluate the variability at different antibiotic concentrations, the pairwise differences in resistance values for all isolates were calculated and the values combined to give a sum total for each particular antibiotic concentration. The concentration with the biggest total was defined as the most informative and selected for further analysis. The informative concentrations were 100 μg mL^-1^ for ampicillin, 5 μg mL^-1^ for chloramphenicol, 1 μg mL^-1^ for kanamycin, 0.5 μg mL^-1^ for norfloxacin, 5 μg mL^-1^ for tetracycline and 0.3 μg mL^-1^ for meropenem.

### Distribution of resistance

**Figure 2:**
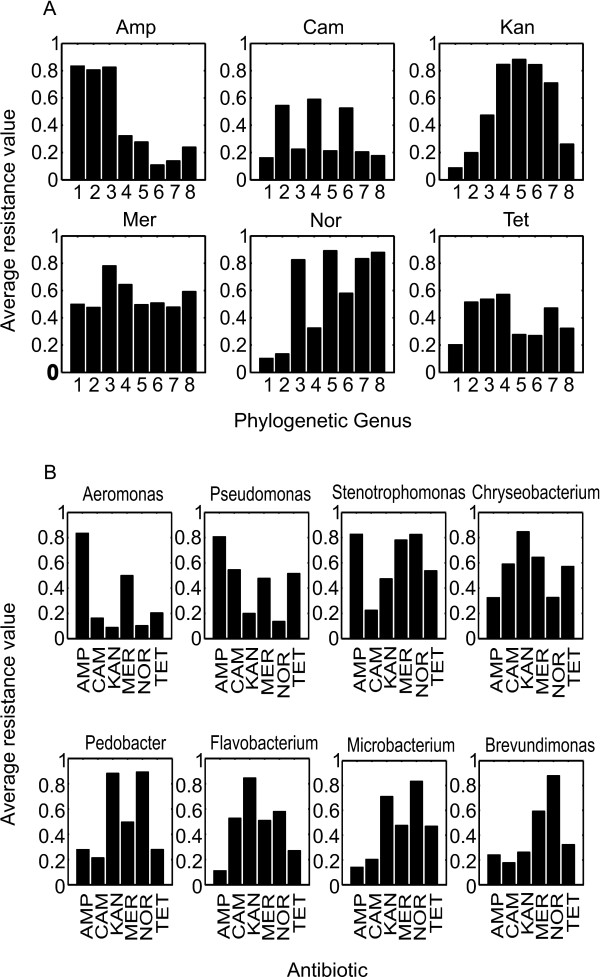
**The average values of resistance coefficients in a specific genus as grouped by antibiotics (A) and genera (B).** (**A**) The genera are organized by antibiotics tested. 1 – *Aeromonas*, 2 – *Pseudomonas*, 3 – *Stenotrpophomonas*, 4 – *Chryseobacterium*, 5 – *Pedobacter*, 6 – *Flavobacterium*, 7 – *Microbacterium*, 8 – *Brevundimonas*. (**B**) The antibiotics tested are organized by genera. Concentrations of the antibiotics were: AMP - ampicillin 100 μg mL^-1^, CAM - chloramphenicol 5 μg mL^-1^, KAN - kanamycin 1 μg mL^-1^, MER - meropenem 0.3 μg mL^-1^, NOR - norfloxacine 0.5 μg mL^-1^ and TET - tetracycline 5 μg mL^-1^.

We analyzed the prevalence of antibiotic resistance in the eight genera that were represented by more than 20 isolates each: *Aeromonas* with 57 isolates (represented by 3 Operational Taxonomic Units (OTU) as defined by the 16S rRNA sequence types), *Pseudomonas* 217 (7 OTUs), *Stenotrophomonas* 73 (5 OTUs), *Chryseobacterium* 86 (25 OTUs), *Pedobacter* 61 (7 OTUs), *Flavobacterium* 41 (11 OTUs), *Microbacterium* 37 (6 OTUs) and *Brevundimonas* 23 (5 OTUs). The number of OTUs indicates that the actual species richness might be lower than can be estimated from the number of isolates. On the other hand, the similarity of the 16S rRNA sequences is not always a sensitive enough criterion to distinguish different species [38,39]. In most cases, one OTU contains small number of isolates making it impossible to analyze the data at OTU level. Therefore the subsequent analyses (Figure 2) were performed at the level of genus. Still, it is interesting to note that three major OTUs of *Chrysobacterium* had considerably different resistance patterns when compared between each other (Table 1). OTU “A”, containing 18 isolates is considerably more sensitive to ampicillin, meropenem (p value 10^-5^) and norfloxacin when compared to OTU “C”, containing 13 isolates. OTU “B”, containing 11 isolates was more sensitive to ampicillin, meropenem, norfloxacin and tetracycline when compared to OTU “C”. There were no significant differences between “A” and “B”.

**Table 1:** Antibiotic resistance differences between 3 OTUs of *Chryseobacterium* (p-values according to Welch Two Sample *t*-test)

	**A vs B**	**A vs C**	**B vs C**
Ampicillin	0.7901	3.24E-15	1.05E-06
Meropenem	0.9101	1.15E-05	6.50E-04
Norfloxacin	0.3138	2.78E-06	0.0052
Tetracycline	0.1027	0.1219	0.011
Chloramphenicol	0.3386	0.374	0.8194
Kanamycin	0.5435	0.121	0.7245

We found that with every antibiotic some genera were almost completely resistant to the drug (*Aeromonas* to ampicillin), whereas others were quite sensitive (*Flavobacterium* to ampicillin; Figure 2A). The only exception was meropenem, where all of the genera characterized had an average resistance value 0.5 or higher. None of the 6 antibiotics was able to inhibit growth of all isolates significantly in any of the phylogenetic groups.

When we analyzed the data according to the phylogenetic groups, we found that in every group some antibiotics inhibited most of the isolates and some did not inhibit any (Figure 2B). Therefore, some of the resistance might be determined by the phylogenetic affiliation, probably indicating intrinsic resistance mechanisms [4,40].

**Figure 3:**
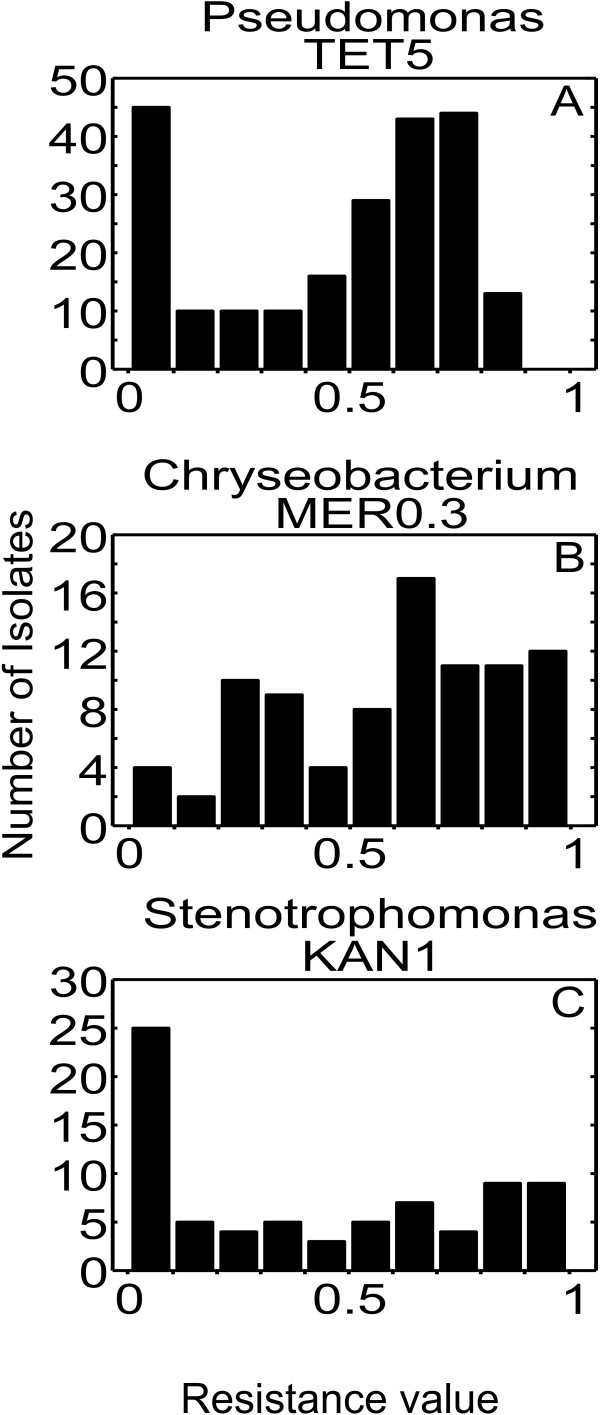
**Examples of resistance coefficient distributions.** Antibiotic abbreviations are as indicated in the legend for Figure 2. The resistance coefficient distributions among the eight most numerous genera on antibiotics where the average resistance value for the genus was between 0.3 and 0.7 are provided as Additional file 1: Figure S1.

Several genera had an average resistance value of around 0.5 (between 0.3 and 0.7). To evaluate whether these average resistance values were caused by the presence of a mixture of fully resistant and fully sensitive isolates, or whether they were caused by an intermediate resistance of all isolates, we analyzed the resistance coefficient distribution within each genus (Figure 3 and Additional file 1**:** Figure S1). In all cases there was a wide distribution of resistance values, although in some cases grouping around the lowest and highest values can be observed (for example the *Pseudomonas* isolates analyzed on tetracycline (Figure 3A)). The highly variable resistance within phylogenetic groups suggests that acquired resistance is responsible for the phenomenon.

### Distribution of multiresistance

Several phylogenetic groups showed a high resistance to more than one antibiotic. This could be due to the existence of “superbugs” that are resistant to many drugs and known to thrive in clinical settings [41]. Alternatively, there might be a random distribution of intrinsic and natural resistance levels.

**Figure 4:**
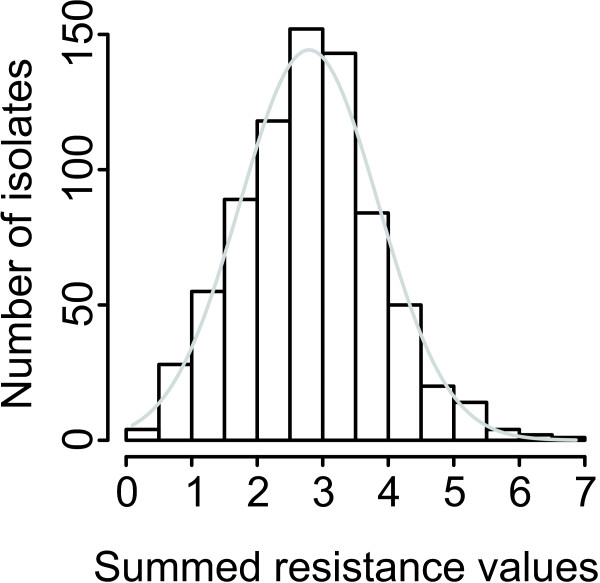
**Distribution of the combined resistance values measured for the six antibiotics used.** The bars indicate the numbers of isolates with combined resistance values in 0.5 increments. The grey line shows a theoretical normal distribution for a population with the same size and mean value.

To test this, we combined the resistance level values for every isolate on all six antibiotics. The results show (Figure 4) that the resistance levels to different drugs demonstrated a normal distribution, which was confirmed by the Kolmogorov-Smirnov test for normality (p = 0.40). This indicates that there is no tendency of the resistance determinants to group together or avoid each other, suggesting that multiresistance happens by chance and that there is no selection for it within the freshwater environment. The existence of multiresistant “superbugs” would manifest itself as a skewed distribution towards the right elbow, but there is no such trend.

It has to be noted that where an isolate is completely resistant to all antibiotics used, the combined value would be 6. The larger values in our dataset indicate uncontrolled fluctuations in OD measurement, or strains able to use the antibiotics for their own benefit [42].

### Resistance correlations

**Figure 5:**
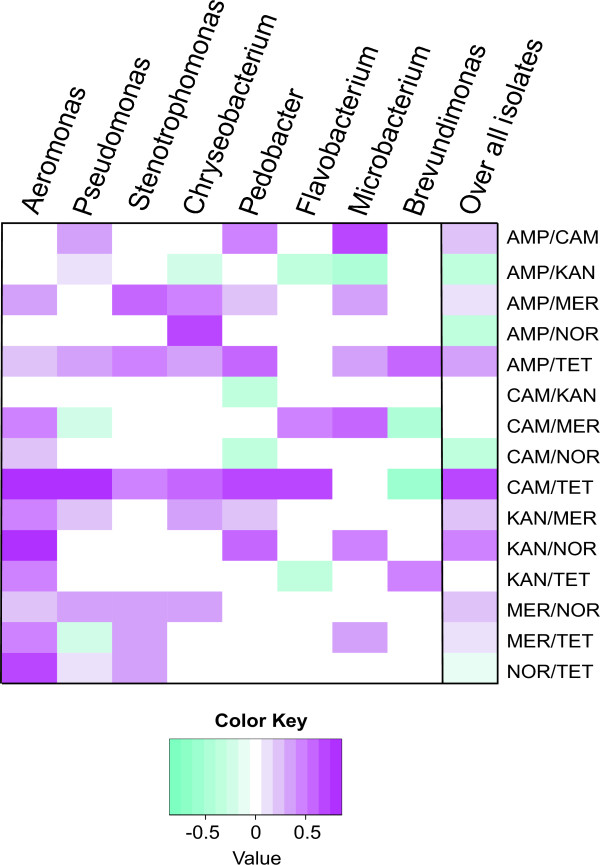
**Heat-map of the correlation coefficients (p-value < 0.05) between the antibiotic pairs.** White cells mean that there was no correlation or that the correlation was statistically not significant (p-value > 0.05). AMP - ampicillin, CAM - chloramphenicol, KAN - kanamycin, MER - meropenem, NOR - norfloxacine and TET - tetracycline.

The apparently random grouping of resistance levels (Figure 4) does not exclude the possibility that some specific resistances group together. To test this we calculated the correlation coefficients for the resistance levels between all antibiotic pairs in the dataset. Eight significant (p < 0.05) positive correlations and four negative correlations were observed (Figure 5). The highest correlation was between tetracycline and chloramphenicol resistance levels, with a correlation coefficient of 0.669 (p < 0.05, N = 760). All of the other correlations were between −0.5 and 0.5 (Figure 5). In addition to the pairwise correlations, we also investigated the possibility of correlations between three antibiotics that would not be explained by the pairwise correlations, but we observed no such correlations.

It is possible that a correlation between resistance levels is caused by a very strong correlation within a specific phylogenetic group, and is not the property of the complete dataset. To analyze this we also calculated the correlations in the eight bigger genera, which contained more than 20 isolates each (Figure 5). A strong positive correlation between tetracycline resistance and chloramphenicol resistance was observed in six of the eight phylogenetic groups analyzed, in case of *Aeromonas* the correlation coefficient being as high as 0.859 (p < 0.05, N = 57). This widespread linking of tetracycline and chloramphenicol resistance suggests that either there is a common multidrug efflux pump active for both compounds [11], or that separate resistance determinants are bundled together into the same plasmid or transposon that is capable of efficient horizontal spread. These molecular mechanisms await further studies.

## Conclusions

The study population which was isolated from river Emajõgi, Estonia did have isolates which were resistant to several antibiotics although the distribution of summed resistances had a normal distribution, which shows that the resistance determinants do not group together or avoid each other. This normal distribution did not mean that there were no correlations between the resistances. The highest correlation was between tetracycline and chloramphenicol resistance.

## Abbreviations

AR: Antibiotic resistance; rRNA: ribosomal RNA; OD: Optical density; MIC: Minimum inhibitory concentration; OTU: Operational taxothe manuscript and participated in the coordination of the study.

## Authors’ contributions

VV, VK and TT conceived the study. VV collected the strains and performed resistance measurements. VV and AJ analyzed the data. VV, AJ, VK and TT wrote the paper. All authors read and approved the final manuscript.

## Supplementary Material

Additional file 1 **Figure S1.** Resistance coefficient distributions among the 8 most numerous genera on antibiotics where the genus’s average resistance value was between 0.3 and 0.7. Click here for file
