# Supplementary material for: Co-occurrence of resistance to different antibiotics among aquatic bacteria
Source: BMC Microbiol. 2012 Oct 2;12:225. doi: 10.1186/1471-2180-12-225 (PMC3519559; doi:10.1186/1471-2180-12-225)
Supplement: Additional file 1 — Figure S1. Resistance coefficient distributions among the 8 most numerous genera on antibiotics where the genus’s average resistance value was between 0.3 and 0.7. [file 1471-2180-12-225-S1.doc]

Supplemental material:

Supplental figure S1


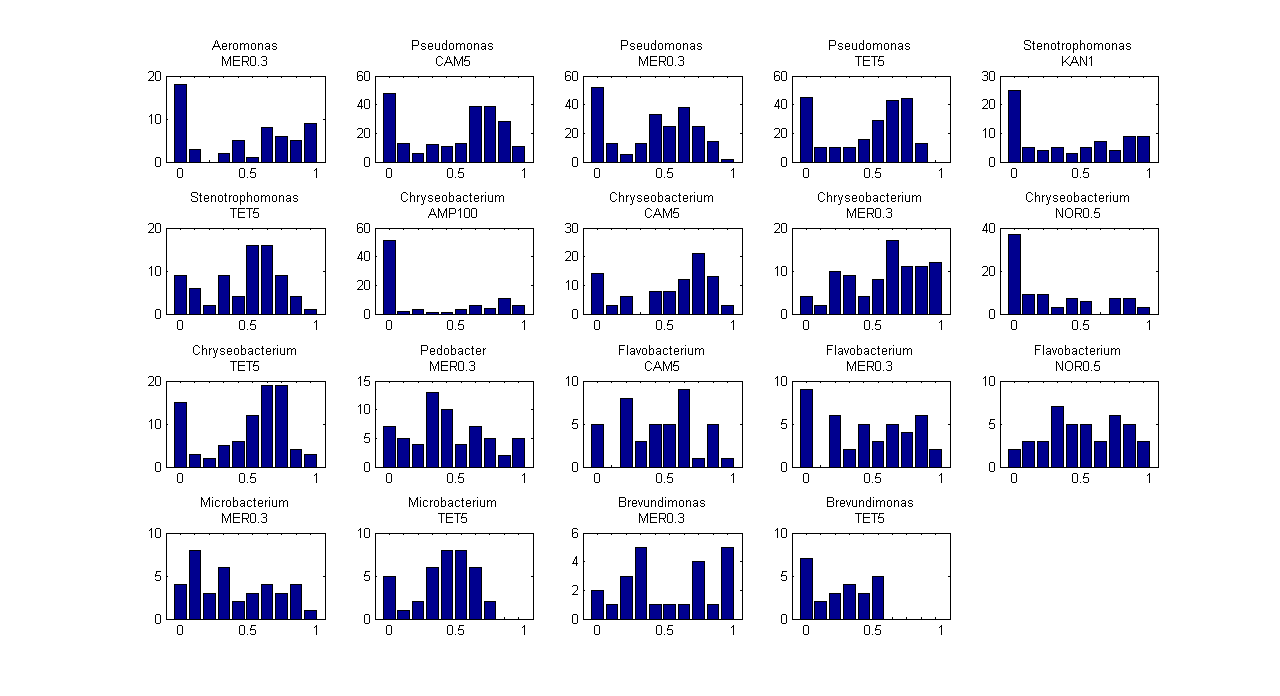


**Figure S1: -** Resistance coefficient distributions among the 8 most numerous genera on antibiotics where the genus’s average resistance value was between 0.3 and 0.7.
